# Complete Genome Sequence of a Novel Putative RNA Virus, RiPV-2, from the Bean Bug Riptortus pedestris

**DOI:** 10.1128/MRA.01584-19

**Published:** 2020-05-21

**Authors:** Yi-Ting Yang, Se Jin Lee, Yu-Shin Nai, Jae Su Kim

**Affiliations:** a Department of Agricultural Biology, College of Agriculture & Life Sciences, Chonbuk National University, Jeonju, Republic of Korea; b Department of Microbiology and Cell Science, University of Florida, Gainesville, Florida, USA; c Department of Entomology, National Chung Hsing University, Taichung, Taiwan; KU Leuven

## Abstract

A novel putative single-stranded RNA virus was discovered from the transcriptome of a bean bug, Riptortus pedestris, infected with the entomopathogenic fungus Beaveria bassiana JEF-007. The complete genome sequence was 9,915 nucleotides long and encoded a 2,916-amino-acid polyprotein. This virus belonged to *Iflaviridae* based on phylogenetic analysis and was named RiPV-2.

## ANNOUNCEMENT

Riptortus pedestris (Hemiptera: Alydidae) is a serious soybean pest in the Republic of Korea and Japan (1, 2). Beauveria bassiana is an entomopathogenic fungus which could be developed as an eco-friendly commercial myco-insecticide for biological control (2). To understand gene regulations during fungal infection, a transcriptome comparison between B. bassiana JEF-007-infected and noninfected *R. pedestris* was performed (2). The preparation of sequencing libraries was done using the TruSeq RNA library preparation kit v2 (Illumina, San Diego, USA). The two libraries were then sequenced using an Illumina HiSeq 2000 instrument (2). The raw sequencing reads (74,595,278 and 78,995,840 reads from B. bassiana JEF-007-infected and -noninfected *R. pedestris* bean bugs, respectively) were filtered using FastQC, and adaptors were trimmed using Trimmomatic (3). The trimmed reads were subjected to *de novo* assembly using the Trinity assembler (4). All the obtained contigs were subjected to an NCBI blastx search, and two novel small RNA viral contigs were found in the B. bassiana JEF-007-infected *R. pedestris* library, showing a low identity (<50%) to known viruses. One of the viral sequences was previously characterized and named RiPV-1 in 2016 (1). Using the second novel small RNA viral contig as a reference genome, 12,865 reads from the entire B. bassiana JEF-007-infected *R. pedestris* library were mapped against this contig using Geneious v9.0.5 (5).

The second assembled viral sequence (c36050_g1_i1; 9,915 bp) was subjected to an NCBI blastx search, and the first hits were Hubei picorna-like virus 33, which is an unclassified RNA virus with 46.2% identity (6), followed by an unknown protein of *R. pedestris* containing a picornavirus capsid protein domain (7) and a polyprotein of Diaphorina citri picorna-like virus with 29.1% identity. Therefore, this viral sequence reveals a novel small positive single-stranded RNA (ssRNA) insect virus of bean bugs named RiPV-2.

**FIG 1:**
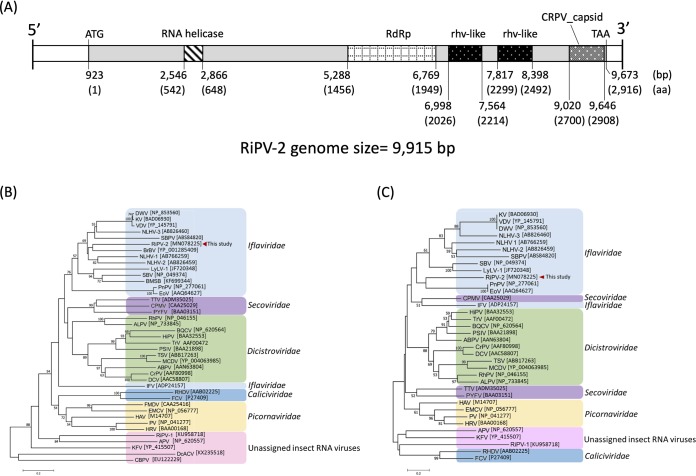
Genome and phylogeny of RiPV-2. (A) Genomic map of RiPV-2. RdRp, RNA-dependent RNA polymerase. Numbers in parentheses are amino acids. (B and C) Phylogenetic analyses of putative RdRp domains (B) and putative helicase domains (C). The neighbor-joining trees were produced and bootstrapped (1,000 replicates) using MEGA7 software; multiple alignments of protein domains were obtained using CLUSTAL_X (11) and edited in GeneDoc (12).

The size of the RiPV-2 RNA genome is 9,915 nucleotides (nt) with a coverage of 371× and a GC content of 36%. Both the 5′ and 3′ ends were confirmed using the rapid amplification of cDNA ends (RACE) technique (8, 9). The 5′ and 3′ untranslated regions (UTRs) of RiPV-2 were 922 and 242 nt, respectively. The genome of RiPV-2 was analyzed using NCBI ORFfinder, and proteins were annotated using NCBI BLASTp with the database of nonredundant (nr) protein sequences. Only one open reading frame (ORF) was predicted in the RiPV-2 genomic RNA, which ranged from 923 to 9,673 nt, encoding a putative polyprotein of 2,916 amino acids. The RNA helicase (Pfam entry pfam00910) and RNA-dependent RNA polymerase (RdRp) (Pfam entry pfam00680) domains were found at amino acid positions 542 to 648 and 1456 to 1949, respectively. Two structural domains were identified as rhv-like (cd00205, picornavirus capsid protein domain-like) motifs at positions 2026 to 2214 and 2299 to 2492, respectively. Another structural protein was predicted as CRPV_capsid (Pfam entry pfam08762, CRPV capsid protein like) motifs at position 2700 to 2908 (Fig. 1).

The RdRp amino acid sequence of RiPV-2 showed the highest-similarity score (95∼99% query coverage) with Hubei picorna-like virus 33 (6), Shahe heteroptera viruses 2 and 1 (6), Brevicoryne brassicae virus-UK, and Darwin bee virus 3, with identities of 64%, 45%, 44%, 41%, and 40%, respectively. The phylogenetic analysis based on the RdRp motif and helicase motif revealed that RiPV-2 represents a distinct species within the genus *Iflavirus*, closely related to Bovine rhinitis B virus (BrBV), Perina nuda virus (PnV), and Ectropis obliqua picorna-like virus (EoV) (Fig. 1), while the nonstructural and structural domain arrangement in the polyprotein was similar to that of *Marnaviridae* (10).

In conclusion, the sequence of RiPV-2 represents a novel RNA virus with distinct genomic characteristics compared to RiPV-1, which was found in the same insect host, *R. pedestris*, and belongs to unclassified *Picornavirales*.

### Data availability.

The complete genome sequence of the RiPV-2 genomic RNA has been deposited in GenBank under the accession number MN078225. The raw data have been deposited in the NCBI Sequence Read Archive (SRA) under the accession number SRP068540.
